# Case of Membranous Croup—New Method of Treatment—Recovery

**Published:** 1877-07

**Authors:** Alexander Fulton

**Affiliations:** Conshohocken, Pa.


### Case of Membranous Croup—New Method of Treatment—Recovery.

By Alexander Fulton, M. D., of Conshohocken, Pa.

On the evening of the 22d of last month I was called in great haste to see a child of W. L. Found complaint membranous croup, of which the child was apparently dying; skin cold and clammy; pulse rapid and thready; face pallid; eyes sunken, half open and fixed, and the breathing very difficult, with that crowing noise so peculiar to the affection; suffocation seemingly imminent.

Having had a number of cases of this terrible disease, all of which proved fatal, notwithstanding careful treatment according to our text books, I determined putting into effect a new procedure, which I had contemplated doing in the very next case that presented itself, viz: I introduced my little finger into the child's mouth, over the tongue, until the epiglottis was reached, then pushed it into the larynx, as I supposed, and still forward, whether between or beyond the vocal cords I do not know. Directly, the child took violent fits of spasmodic coughing, followed immediately by the elimination of large mouthfuls of membranous exudation—very ropy—could be drawn like the white of an egg. The result was, the child on the very threshold of death, became animated; the complexion almost natural; the eyes, that were half opened and fixed, opened; and the breathing became less difficult. Relief was experienced until the next morning, when another paroxysm threatened. Again, I went through the same procedure, followed by the same good result, and prescribed the following, as recommended by Dr. Thomas Drysdale in a former issue of the *Reporter*:—

℞. Pulv. potassæ chlor., ʒij

Syrup limon, f.℥j

Aquæ, f.℥iij. M.

Sig.—A teaspoonful every hour.

Convalescence ensued, with complete recovery.—*Medical and Surgical Reporter.*

